# Amide Proton Transfer Imaging of Diffuse Gliomas: Effect of Saturation Pulse Length in Parallel Transmission-Based Technique

**DOI:** 10.1371/journal.pone.0155925

**Published:** 2016-05-26

**Authors:** Osamu Togao, Akio Hiwatashi, Jochen Keupp, Koji Yamashita, Kazufumi Kikuchi, Takashi Yoshiura, Masami Yoneyama, Marijn J. Kruiskamp, Koji Sagiyama, Masaya Takahashi, Hiroshi Honda

**Affiliations:** 1 Department of Clinical Radiology, Graduate School of Medical Sciences, Kyushu University, 3-1-1 Maidashi Higashi-ku, Fukuoka, 812–8582, Japan; 2 Philips Research Europe, Röntgenstrasse 24–26, Hamburg, 22335, Germany; 3 Philips Electronics Japan, 2-13-37 Konan Minato-ku, Tokyo, 108–8507, Japan; 4 Advanced Imaging Research Center, UT Southwestern Medical Center, 2201 Inwood Rd, Dallas, Texas, 75235, United States of America; Brighton and Sussex Medical School, UNITED KINGDOM

## Abstract

In this study, we evaluated the dependence of saturation pulse length on APT imaging of diffuse gliomas using a parallel transmission-based technique. Twenty-two patients with diffuse gliomas (9 low-grade gliomas, LGGs, and 13 high-grade gliomas, HGGs) were included in the study. APT imaging was conducted at 3T with a 2-channel parallel transmission scheme using three different saturation pulse lengths (0.5 s, 1.0 s, 2.0 s). The 2D fast spin-echo sequence was used for imaging. Z-spectrum was obtained at 25 frequency offsets from -6 to +6 ppm (step 0.5 ppm). A point-by-point B0 correction was performed with a B0 map. Magnetization transfer ratio (MTR_asym_) and ΔMTR_asym_ (contrast between tumor and normal white matter) at 3.5 ppm were compared among different saturation lengths. A significant increase in MTR_asym_ (3.5 ppm) of HGG was found when the length of saturation pulse became longer (3.09 ± 0.54% at 0.5 s, 3.83 ± 0.67% at 1 s, 4.12 ± 0.97% at 2 s), but MTR_asym_ (3.5 ppm) was not different among the saturation lengths in LGG. ΔMTR_asym_ (3.5 ppm) increased with the length of saturation pulse in both LGG (0.48 ± 0.56% at 0.5 s, 1.28 ± 0.56% at 1 s, 1.88 ± 0.56% at 2 s and HGG (1.72 ± 0.54% at 0.5 s, 2.90 ± 0.49% at 1 s, 3.83 ± 0.88% at 2 s). In both LGG and HGG, APT-weighted contrast was enhanced with the use of longer saturation pulses.

## Introduction

Chemical exchange saturation transfer (CEST) has attracted great attention in the area of molecular imaging as a novel contrast mechanism in MR imaging [1]. CEST contrast is obatined by applying a saturation pulse at the resonance frequency of a slow-intermediate exchanging proton site (-NH, -OH, -H_2_O) of endogenous or exogenous agents, and the resulting saturated or partially saturated protons are transferred to bulk water via chemical exchange [1–4]. The CEST effect is to reduce the signal intensity of bulk water detected in the proton MR imaging, thereby providing negative contrast in obtained images. The low concentration solutes with exchangeable protons are detected indirectly through the water signal with greatly enhanced sensitivity [3].

Amide proton transfer (APT) imaging is one of the endogenous CEST imaging methods developed by the group of Zhou and van Zijl et al. [5]. APT imaging exploits the exchange between the amide protons (-NH) in endogenous mobile proteins and bulk-water protons [5]. The APT-weighted signal quantified by the asymmetry of the magnetization transfer (MT) at +3.5 ppm relative to bulk water is considered to reflect tissue concentration of amide proton contained in proteins/peptides, which are significantly enhanced in active tumors [6]. APT imaging is expected to play an important role in imaging of diffuse gliomas [7]. Recently, it has been demonstrated that APT imaging is useful in predicting histopathological grades of diffuse gliomas in patients [8]. It has also been shown that APT imaging could monitor therapeutic response to chemotherapy in a mouse model of human glioblastoma multiforme (GBM) [9], and in discrimination of radiation necrosis from active glioma in a rat model [10].

Since CEST signal depends upon power and length of radio frequency (RF) saturation pulse, optimization is a key factor for sensitivity of CEST imaging. Many CEST research studies have been performed on animal MRI systems with small-sized bore and very different design of the RF power transmission chain [6, 11, 12]. In such systems, long RF saturation pulses (typically 5–10 s) is usually readily available by continuous wave pulse without any special modifications in the saturation pulses. On the other hand, in clinical scanners, the length of saturation pulses is limited (typically < 1 s) due to restrictions of specific absorption rate (SAR) and RF duty cycle. Recently, a method based on parallel RF transmission has been developed, which allows for the use of arbitrarily long RF pulses in any types of imaging sequence on clinical scanners via RF amplifier alternation within the SAR and RF duty cycle limits [13–15]. Therefore, we tested how APT-weighted signal depends upon saturation length in clinical settings.

## Materials and Methods

### Patients

This study was approved by the institutional review board of Kyushu University Hospital (the approval number 23–125), and written informed consent was obtained. Twenty-two adult patients with diffuse glioma (46.1 ± 13.8 years old, 9 males and 13 females) who underwent subsequent surgical resection were included in this prospective study. The number of patients of low-grade glioma was 9 (5 diffuse astrocytoma, 3 oligodendroglioma, 1 oligoastrocytoma) and that of high-grade glioma was 13 (3 anaplastic oligodendroglioma, 1 anaplastic oligoastrocytoma, 9 GBM). All tumors were located in the supratentorial region of the brain. The interval between MR imaging and surgery was shorter than 2 weeks in all patients.

### MR Imaging

MR imaging was conducted on a 3T clinical scanner (Achieva 3.0TX, Philips Healthcare, Best, The Netherlands) equipped with second-order shims, using an 8-channel head coil for signal reception and 2-channel parallel RF transmission via the body coil. The alternation of the two transmission channels enables long quasi-continuous RF saturation beyond the 50% duty-cycle of a single RF amplifier [16]. Parallel transmission-based APT imaging functionally allows saturation pulse lengths of up to 5 s with the full available power of one RF amplifier for the maximum B1 field amplitude. Since all imaging pulses within a parallel transmission-based APT sequence are using both amplifiers together in a standard way, there are no restrictions regarding the choice of MR sequence types (spin-echo/gradient-echo). In addition, special RF shimming for the saturation homogeneity of the alternated pulse was applied [16].

Two-dimensional (2D) APT imaging was performed on a single slice delineating the maximum cross-section area of a tumor. We used a saturation pulse (B1 = 2μT) with various durations, 0.5, 1 and 2s that was determined by the number of applied 50ms sinc-gauss-sharped element, 10, 20 and 40, respectively. To obtain a Z-spectrum, imaging was repeated at 25 saturation frequency offsets from ω = −6 to +6 ppm with a step of 0.5 ppm as well as one far off-resonant frequency (ω = −1560 ppm) for signal normalization. The other imaging parameters were as follows: fast spin-echo readout with driven equilibrium refocusing; echo train length 128 (single shot FSE); repetition time (TR) = 5,000 ms; echo time (TE) = 6 ms; Matrix = 128 × 128 (reconstructed to 256 × 256); slice thickness = 5 mm, field of view = 230 × 230 mm; scan time = 2 min 20 s for one Z-spectrum. A B0 map for off-resonance correction was acquired separately using a 2D gradient-echo (TR = 15 ms, TE = 8.1 ms, dual echo, ΔTE = 1 ms, 16 averages, 33 s) with identical spatial resolution, and it was used for a point-by-point B0 correction. For reference, several standard MR images, including T1-weighted, T2-weighted, fluid attenuation inversion recovery (FLAIR), and contrast enhanced T1-weighted images were acquired. The APT images were performed before administration of gadolinium contrast agent in all patients since T1 shortening by gadolinium may alter APT-weighted signal.

### APT Imaging Data Analysis

Analysis of APT imaging was performed with the software program ImageJ (version 1.43u; National Institutes of Health, Bethesda, MD). A dedicated plug-in was build to analyze the Z-spectra and asymmetry of magnetization transfer ratio (MTR_asym_) equipped with a correction function for B0 inhomogeneity as previously demonstrated [8, 14]. Briefly, the local B0 field shift in Hz was obtained from the B0 map. The B0 map was created from dual-echo gradient-echo images (ΔTE = 1 ms) according to the following equation;

B0(x)={Phase[TE2](x)−phase[TE1](x)}/[(TE2−TE1)*2*Pi],

where Phase [TEi](x) indicates image phases of the images with echo times TE1 or TE2 at position x in radian, and B0(x) is the resulting B0 map measured in Hz. Each voxel was corrected in image intensity for the nominal saturation frequency offset by Lagrange interpolation among the neighboring Z-spectral images. This procedure corresponds to a frequency shift along the saturation frequency offset axis according to the measured B0 shift.

The Z-spectrum was calculated as S_sat_/S_0_, where S_sat_ and S_0_ are the signal intensity obtained with and without selective saturation, respectively [5]. To reduce these undesired contributions from conventional MT effect and direct saturation of bulk water, an asymmetry analysis of Z-spectrum with respect to the water frequency was performed as MTR_asym_.

$${MTR}_{asym}=\frac{S_{sat}(-\text{offset})-S_{sat}(+\text{offset})}{S_{0}}$$

APT-weighted signal is the asymmetry of the Z-spectrum at 3.5 ppm calculated as MTR_asym_ (3.5 ppm).

$$APT\;signal={MTR}_{asym}\;(3.5\;ppm)=\frac{S_{sat}(-3.5\;ppm)-S_{sat}(+3.5\;ppm)}{S_{0}}$$

Signal intensity was measured in circular region-of-interests (ROIs, approx. 0.6 cm^2^, 72 pixels) independently by two trained neuroradiologists (K.Y. and O.T., 14 and 16 years of experience in neuroradiology, respectively). Then one to five ROIs were placed depending on the lesion size in the solid component of a tumor and the best effort was given to avoid cystic, necrotic, or hemorrhagic components of the tumor with reference to conventional MR images. The same ROIs were used in the images for the three saturation pulse length in each patient. The average of the measurements in the ROIs was calculated to represent each tumor. The signal was also measured in a ROI placed in normal appearing white matter (NAWM).

### Pathological Evaluation

The pathological diagnosis was determined with specimens removed at surgical resection according to the World Health Organization (WHO) criteria by established neuropathologists.

### Statistical Analysis

All values are expressed as mean ± standard deviation (SD). MTR_asym_ (3.5 ppm) was measured in tumor as well as in NAWM. ΔMTR_asym_ (3.5 ppm) was defined as the difference in MTR_asym_ (3.5 ppm) between tumor and NAWM. The inter-observer agreement regarding the measurements for MTR_asym_ (3.5 ppm) or ΔMTR_asym_ (3.5 ppm) by the two observers was analyzed by calculating the intra-class correlation coefficient (ICC). ICCs are considered to be excellent if > 0.74. The measurements by the observer 1 were used for further statistical analyses when the ICC were excellent. In the patient groups of low-grade glioma (LGG, Grade II) and high-grade glioma (HGG, Grade III and IV), MTR_asym_ (3.5 ppm) of tumor and ΔMTR_asym_ (3.5 ppm) were compared among the three saturation pulse lengths by one-way analysis of variance followed by Tukey’s multiple comparison test. MTR_asym_ (3.5 ppm) of tumor and ΔMTR_asym_ (3.5 ppm) were compared between LGG and HGG for each saturation pulse length by Student’s t-test. Statistical analyses were performed with a commercially available software package (SPSS, IBM 19, Armonk, NY, or Prism 5.0, GraphPad Software, Inc., San Diego, CA). *P*-values < .05 were considered significant.

## Results

### Inter-observer Agreement

The ICCs for the MTR_asym_ (3.5ppm) with the 500 ms, 1s, and 2s saturations were 0.954, 0.949, and 0.956, respectively. The ICCs for the ΔMTR_asym_ (3.5ppm) with the 500 ms, 1s, and 2s saturations were 0.908, 0.963, and 0.930, respectively. The inter-observer agreements were considered excellent in all measurements. The measurements of the MTR_asym_ (3.5 ppm) ΔMTR_asym_ (3.5 ppm) by the two observers were showed in S1 Table.

### Z-spectrum

**Fig 1:**
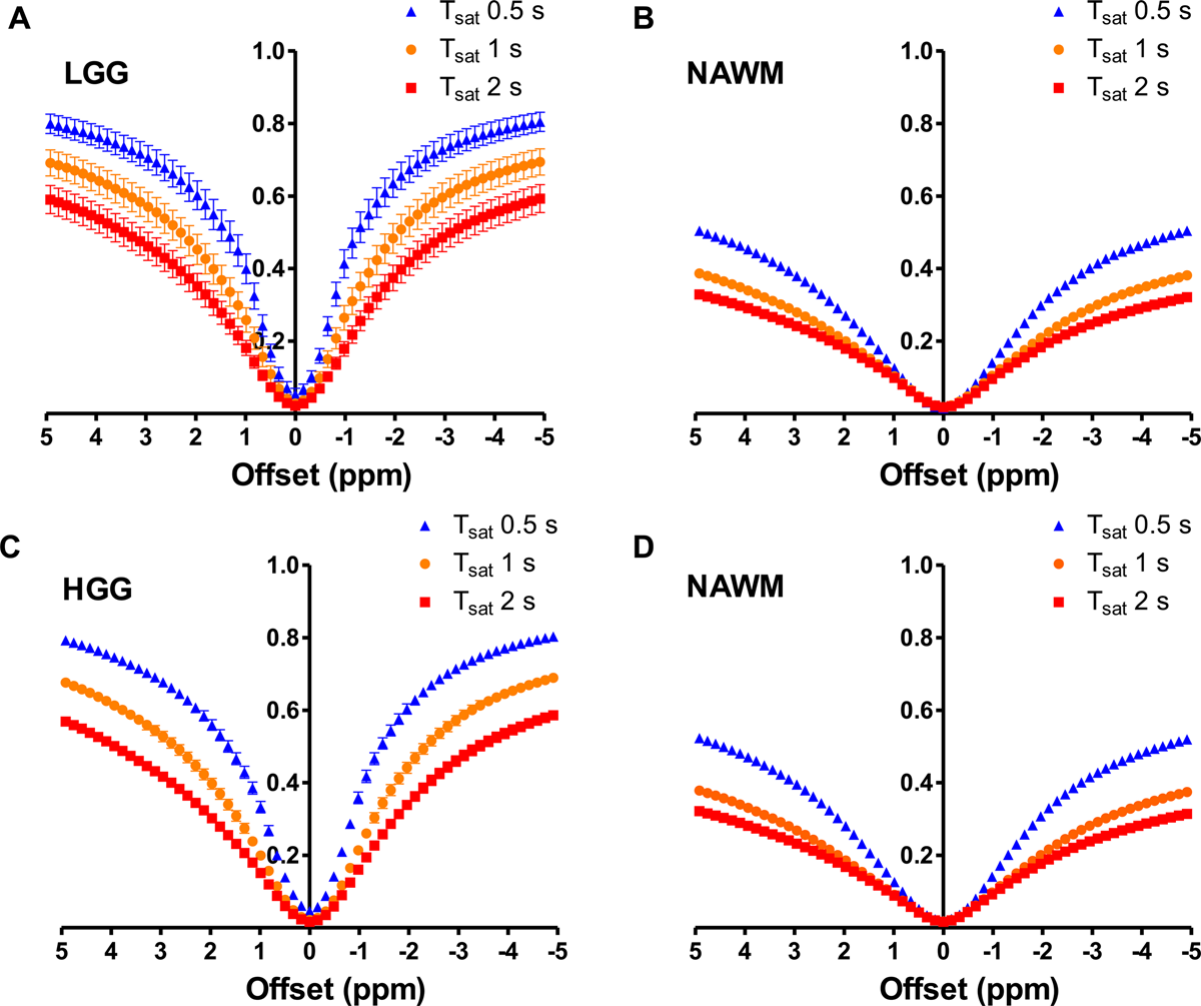
Z-spectra of LGG **(A)**, HGG **(C)**, and corresponding NAWM **(B, D)**. Z-spectra of tumor was steeper than that of NAWM, presumably because of less MT effect in tumor compared with NAWM. Prolongation of saturation pulses results in larger MT effect and thus wider Z-spectra in both tumor and NAWM.

The Z-spectra of LGG (n = 9), HGG (n = 13), and corresponding NAWM (n = 9 and 13, respectively) are shown in Fig 1. In both LGG and HGG, Z-spectrum of tumor was steeper than that of NAWM, indicating less magnetization transfer (MT) effect in tumor compared with NAWM. Increase of saturation pulse length resulted in decreased signal intensity in entire range of Z-spectra in both LGG and HGG as well as in NAWM, which indicated that MT effect and direct saturation became larger in all tissues with longer saturation pulses.

### MTR and ΔMTR asymmetry

**Fig 2:**
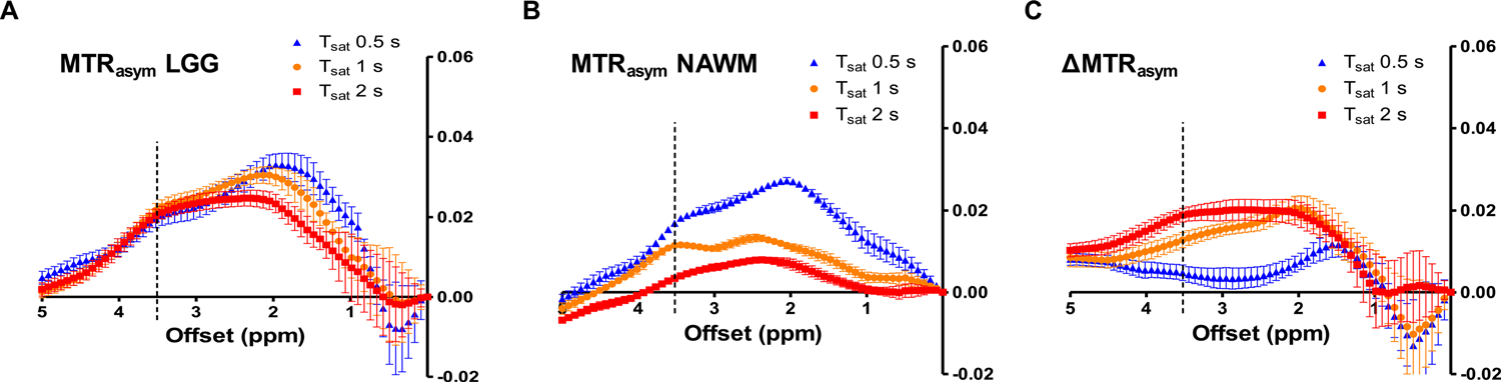
MTR_asym_ of tumor **(A)** and NAWM **(B)** and ΔMTR_asym_ **(C)** in LGG. MTR_asym_ (**A**) of tumor was decreased with the saturation length in lower frequency range (1–2 ppm), but equivalent at 3.5 ppm. MTR_asym_ of NAWM (**B**) is decreased with the saturation length in the entire frequency range. ΔMTR_asym_ (**C**) was increased with the saturation length at higher frequency offsets (>2 ppm).

MTR_asym_ of tumor and NAWM and ΔMTR_asym_ in the patients with LGG are shown in Fig 2. MTR_asym_ of LGG was decreased with the length of the saturation pulse at smaller frequency offsets (1–2 ppm), but almost comparable at around 3.5 ppm. MTR_asym_ of corresponding NAWM was decreased with the length of saturation pulse in the entire frequency range. As a result, ΔMTR_asym_ of LGG was increased with the length of saturation at larger frequency offsets (>2 ppm) including 3.5 ppm.

**Fig 3:**
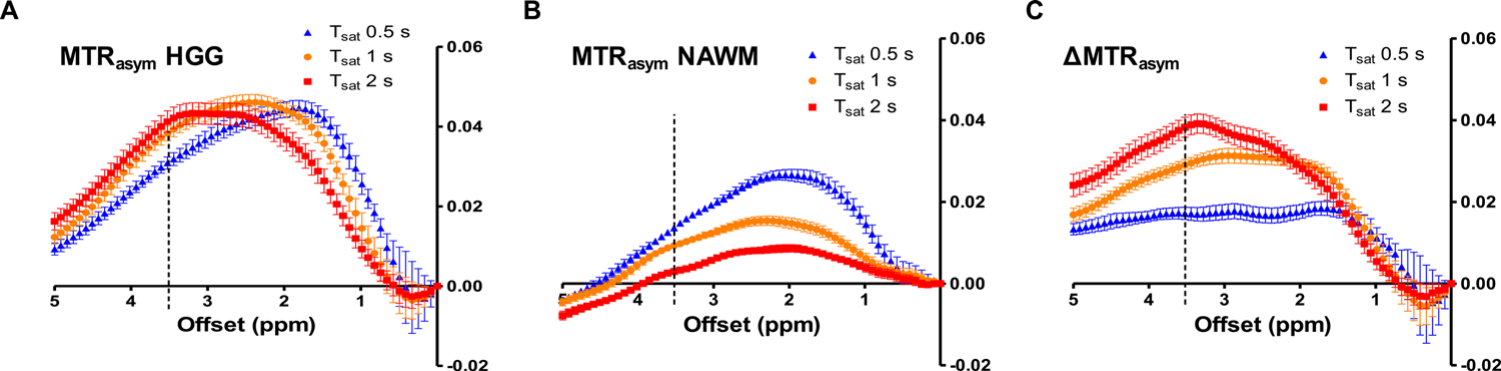
MTR_asym_ of tumor and NAWM and ΔMTR_asym_ in HGG. MTR_asym_ of tumor **(A)** was decreased with the saturation length in lower frequency range (<2 ppm), but was increased at 3.5 ppm. MTR_asym_ of NAWM **(B)** was decreased with the saturation length in entire frequency range. ΔMTR_asym_ **(C)** was increased with the saturation length at higher frequency (>2 ppm). ΔMTR_asym_ (3.5 ppm) with the 2 s saturation reached maximum at around 3.5 ppm (specific frequency of amide protons).

MTR_asym_ of tumor and NAWM and ΔMTR_asym_ in the patients with HGG are shown in Fig 3. MTR_asym_ of HGG was decreased with the length of the saturation pulse in smaller frequency offsets (<2 ppm), but was increased at larger frequency offsets (>2 ppm) including 3.5 ppm. MTR_asym_ of corresponding NAWM was decreased with the length of saturation pulse in the entire frequency range as consistent with the result in the patients with LGG (Fig 2). As a result, ΔMTR_asym_ of HGG was also increased with the length of saturation at larger frequency offsets (>2 ppm). In particular, ΔMTR_asym_ (3.5ppm) with the 2 s saturation reached maximum at approximately 3.5 ppm, which corresponds to the specific frequency of amide protons.

### MTR and ΔMTR asymmetry at 3.5 ppm

**Fig 4:**
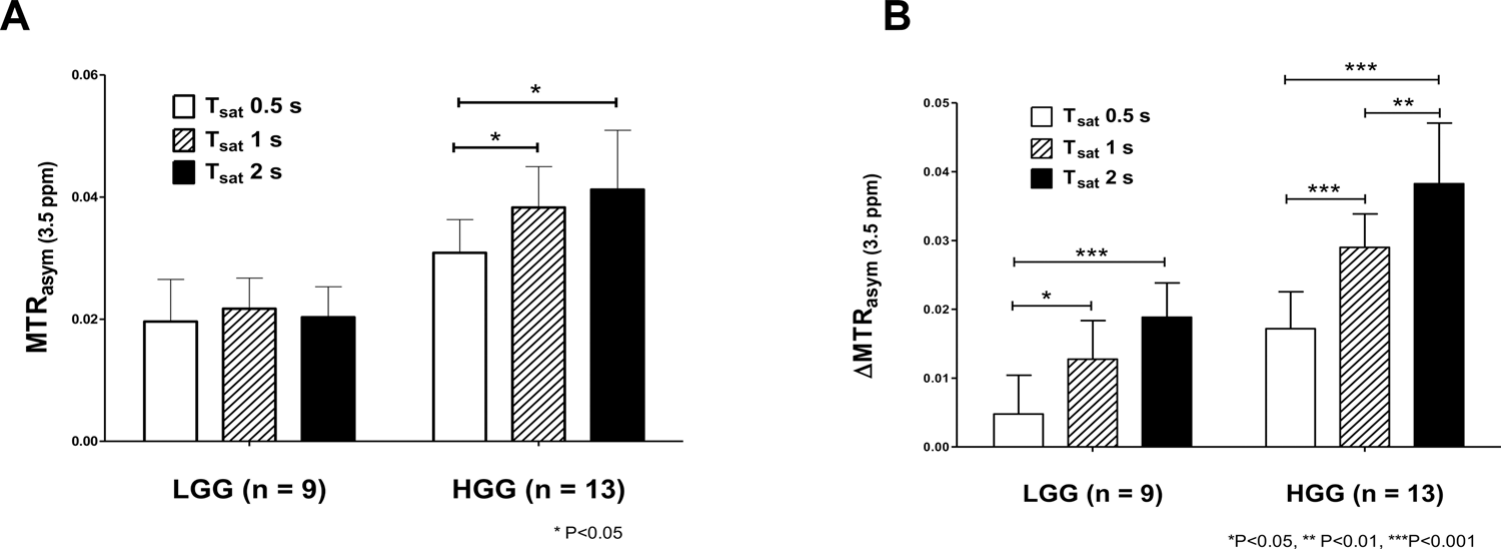
MTR_asym_ (3.5 ppm) and ΔMTR_asym_ (3.5 ppm) of LGG and HGG. No significant differences were observed in MTR_asym_ (3.5 ppm) among the three saturation lengths in LGG, while MTR_asym_ (3.5 ppm) with the 1 s and 2 s saturation was significantly higher than that with the 0.5 s saturation in HGG (**A**). ΔMTR_asym_ (3.5 ppm) with the 1 s and 2 s saturation length was significantly higher than that with the 0.5 s saturation in LGG, and ΔMTR_asym_ (3.5ppm) of HGG significantly increased with the saturation length (**B**). Both MTR_asym_ (3.5ppm) and ΔMTR_asym_ (3.5ppm) were significantly higher in HGG than in LGG at any saturation pulse lengths.

**Fig 5:**
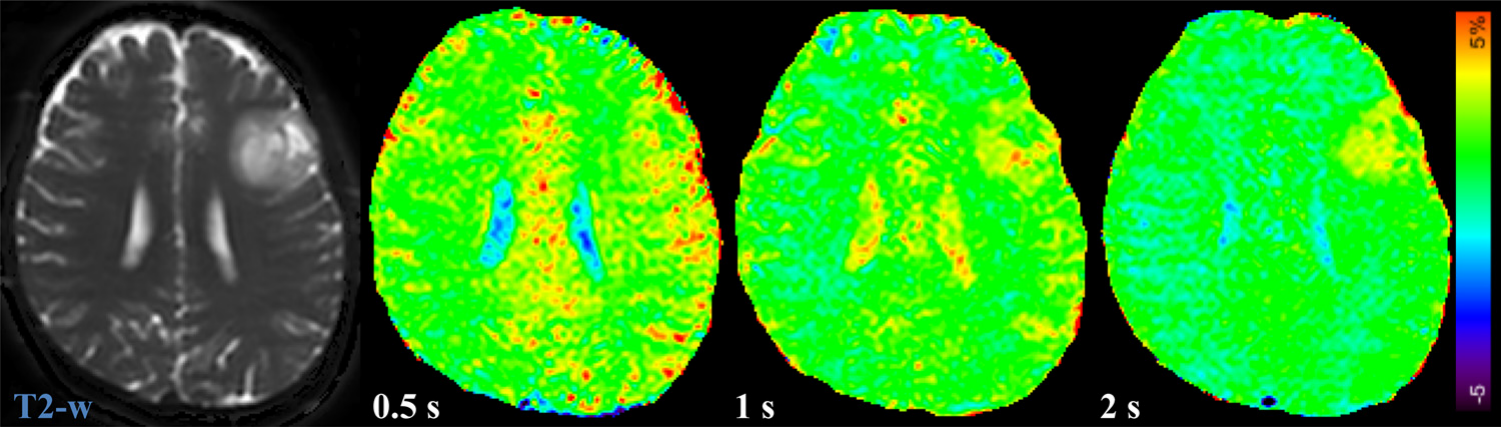
A case of diffuse astrocytoma (Grade II, LGG). The APT-weighted signal of the tumor in the left frontal lobe is almost comparable in all the saturation pulse lengths, but the contrast between tumor and normal brain tissue is slightly increased at longer saturation pulses due to decreased signal in NAWM.

**Fig 6:**
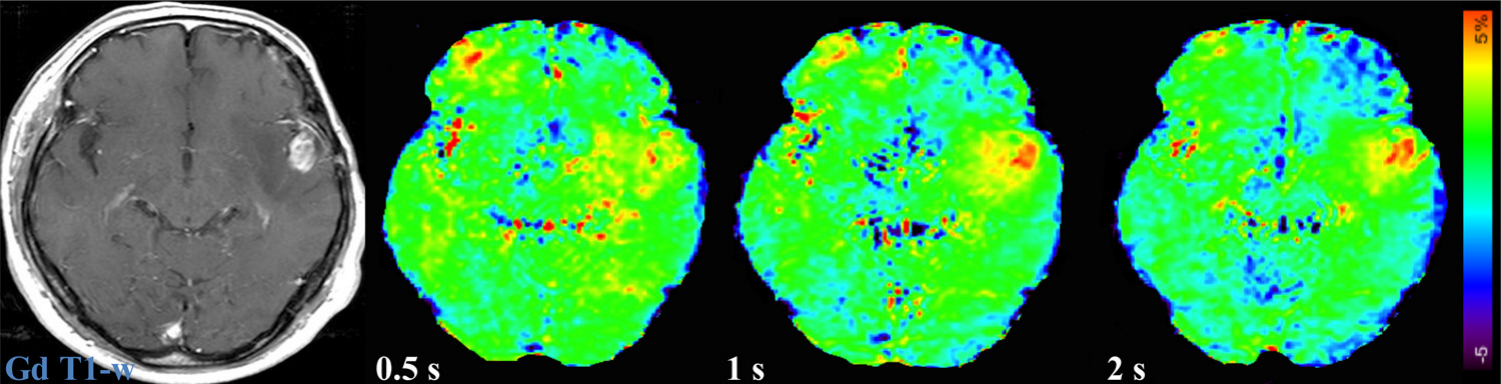
A case of glioblastoma multiforme (Grade IV, HGG). The APT-weighted signal of the tumor in the left temporal lobe is increased with the saturation length, and the contrast between tumor and normal brain tissue becomes larger at longer saturation pulses.

MTR_asym_ (3.5ppm) and ΔMTR_asym_ (3.5ppm) of LGG and HGG are shown in Fig 4. MTR_asym_ (3.5ppm) was not significantly different among the three saturation pulse lengths in LGG (1.96 ± 0.69% at 0.5 s, 2.17 ± 0.50% at 1 s, 2.03 ± 0.50% at 2 s). On the other hand, MTR_asym_ (3.5ppm) with the 1s (3.83 ± 0.67%, P < 0.05) and 2 s (4.12 ± 0.97%, P < 0.05) saturation was significantly higher than that with the 0.5 s (3.09 ± 0.54%) saturation in HGG. ΔMTR_asym_ (3.5ppm) with the 1 s (1.28 ± 0.56%, P < 0.05) and 2 s (1.88 ± 0.56%, P < 0.001) saturation was significantly higher than that with the 0.5 s saturation (0.48 ± 0.56%) in LGG. ΔMTR_asym_ (3.5ppm) of HGG significantly increased with the saturation pulse length (1.72 ± 0.54% at 0.5 s, 2.90 ± 0.49% at 1 s, 3.83 ± 0.88% at 2 s, 0.5 s vs. 1 s, P < 0.001, 1 s vs. 2 s, P < 0.01, 0.5 s vs. 2 s, P < 0.001). Both MTR_asym_ (3.5ppm) and ΔMTR_asym_ (3.5ppm) were significantly higher (P < 0.0001 for all comparisons) in HGG than in LGG with all the three saturation pulse lengths. Fig 5 shows a representative case of LGG. The APT-weighted signal of the tumor was almost unchanged with the saturation pulse length, but contrast between tumor and normal brain tissue was improved at longer saturation pulses due to decreased signal in NAWM. Fig 6 shows a representative case of HGG. The APT-weighted signal of the tumor was increased with the length of saturation, and contrast between tumor and normal brain tissue was improved at longer saturation pulses.

## Discussion

The choice of appropriate imaging parameters, especially RF saturation pulse length and power, is crucial in APT imaging. Regarding saturation power, it was reported that the change in saturation power substantially affected MTR_asym_ and ΔMTR_asym_ of brain tumors [17]. In the present study, the effect of saturation pulse length was evaluated on APT imaging of diffuse gliomas by using a parallel transmission-based technique. Basically, longer saturation pulse length should increase the APT effect since it can build up or maintain saturation and enable more accumulation of exchanged protons in bulk water.

Our present study demonstrated that the MTR_asym_ (3.5ppm) was higher in HGG compared with LGG presumably reflecting abundant mobile proteins and peptides, which is consistent with previous study of Togao et al. [8]. The MTR_asym_ (3.5ppm) of HGG significantly increased with an extended length of the saturation pulse while that of LGG was almost unchanged with saturation pulse length. The Z-spectra of NAWM in both cases of HGG and LGG are almost identical in which the MTR_asym_ (3.5 ppm) decreases with an extended length of the saturation pulse. Consequently, ΔMTR_asym_ (3.5 ppm), which is a contrast in MTR _asym_ (3.5ppm) between tumor and NAWM, increased with the length of saturation pulse in both LGG and HGG. According to the previous studies by Zhou et al. [5, 12], MTR_asym_ (3.5 ppm) is affected not only by the APT effect but also by other factors as follows:

$${MTR}_{asym\;(3.5ppm)}={MTR\prime }_{asym\;(3.5ppm)}+APTR$$

$$APTR=\frac{\left[amide\;proton\right]}{\left[water\;proton\right]}∙\frac{\left(1-e^{-R_{1W}∙t_{sat}}\right)}{R_{1W}}K∙10^{pH-pK_{w}}$$

where MTR’_asym_ (3.5 ppm) is the native or conventional MTR asymmetry at 3.5 ppm, APTR is the amide proton transfer ratio, K is the normalized proton exchange rate between the two proton pools, R_1W_ is the relaxivity of water [5]. Based on this equation, MTR_asym_ (3.5 ppm) is composed of APTR and native MTR asymmetry. APTR depends primarily on mobile amide proton concentration and amide proton exchange rate that are related to tissue pH [17]. The source of the native MTR asymmetry is not fully understood, which may contain the inherent asymmetry of the semi-solid macromolecular MT effect and intramolecular and intermolecular nuclear Overhauser effects of aliphatic protons of mobile macromolecules and metabolites [17, 18]. The increase in MTR_asym_ (3.5 ppm) with longer saturation in HGG can be attributed to increase in APTR due to high concentrations of mobile proteins and peptides in tissue. We assume that a possible reason for the lack of this increase in LGG was the reduction of native MTR asymmetry and small increase in APTR due to lower concentrations of mobile proteins and peptides. The effect of the saturation pulse length acted like that of the saturation power level, as shown in Fig 1, in which the spill-over effects by direct saturation of water and semi-solid MT effects increased with the saturation pulse length. The increased semi-solid MT and the spill-over effects due to longer saturation pulses might diminish both APTR and native MTR asymmetry. In NAWM, MTR_asym_ (3.5ppm) was consistently decreasing with longer RF saturation. This reduction could result from larger MT and spill-over effects and decreased native MTR asymmetry caused by the longer saturation pulse. Subtracting MTR_asym_ (3.5 ppm) in NAWM from that of tumor might cancel the decrease in native MTR asymmetry and thus lead to increase in ΔMTR_asym_ (3.5 ppm) in LGG. In HGG, subtracting MTR_asym_ (3.5 ppm) in NAWM from that of tumor might further contribute to the increase in ΔMTR_asym_ (3.5 ppm). Thus, the long saturation pulses improve the sensitivity of APT imaging for detection of amide proton exchange in LGG as well as in HGG.

In the present study, time-interleaved parallel transmission-based APT imaging was conducted with a fast spin-echo sequence using driven-equilibrium refocusing. A previous study showed that fast spin-echo based APT imaging showed superior sensitivity compared to the gradient-echo sequence [16]. In particular, at low protein concentrations, the driven-equilibrium technique offered up to 2-fold enhancement of contrast-to-noise ratio (CNR). The improvement in CNR with fast spin-echo using driven-equilibrium might help detect low-concentrations of amide protons in LGG. Furthermore, the special RF shimming greatly contributed to spatially homogenous saturation effect, and the B0 map created from the dual-echo gradient-echo sequence resulted in accurate correction of B0 inhomogeneity in APT imaging. These might also contribute to improve the detection of such low-concentrations of amide protons.

There are several limitations in the present study. The differences in recovery time could affect the CEST effect when TR was not long enough compared with tissue T1. The variation of recovery time (2.5 s, 3.5 s, 4 s for saturation time of 2 s, 1 s, 0.5 s, respectively, single-shot image readout time of about 0.5 s) in our study does indeed lead to steady-state higher saturation levels for very long T1 tissue types. Assuming the longest T1 of 2 s, this effect may lead to 40% (20%; 16%) higher steady-state saturation levels for saturation time of 2.0 s (1.0s; 0.5s) as compared to a fully relaxed situation with very long TR (e.g. 20s). Thus, our fixed TR of 5 s approach favors the long saturation pulses for very long T1 species, because the steady-state saturation is enhanced with short recovery times. The effect is strongly reduced for shorter T1 values, e.g. for T1 of 1 s: 9% (3%; 1.8%). Thus, the effect is insignificant at moderate T1 values. In the previous study with egg-white phantoms with T1 of 1639.3 ms, the effect of the difference in TR was evaluated using the exact same imaging scheme as the present study, however, no significant differences were found between the CEST effects obtained with different TRs [15]. Optimal saturation parameters are a combination of saturation power and duration, and thus it was desirable to vary both the saturation duration as well as the power. However, it was impossible to perform many scans in limited scan time in patients who underwent many other clinical scans, and thus we decided to monitor just the effect of saturation length with the fixed saturation power. Zhao et al. used the three different saturation power (1, 2, and 3 μT) with the fixed duration of 500 ms, and found that 2 μT was optimal [17]. In their study, a duration of 500 ms was longest allowed for their body coil, however, our parallel transmission-based technique allowed longer saturation pulses without the limitation for duration. Our findings where the 1 s or 2 s saturation improved the APT-weighted contrast compared with the 500 ms saturation at the power of 2 μT should be meaningful. We thought that the image co-registration among APT-weighted images obtained with the three different saturation lengths was not necessary since these images were sequentially acquired in the exact same geometry and field of view in a single session without repositioning, and severe head movements were not observed in any patients. However, there is a possibility that subtle head movements during the scans might have affected the measurements.

## Conclusions

This study demonstrated the utility of long RF saturation pulses, which was enabled by the parallel transmission-based technique, in APT imaging of diffuse gliomas. It was shown that a significant increase in MTR_asym_ (3.5 ppm), which is the APT-weighted signal, of HGG when the length of saturation pulse became longer, but not in LGG. ΔMTR_asym_ (3.5 ppm), which is the APT-weighted contrast, increased with the length of saturation pulse in both LGG and HGG. The long saturation pulse enabled by this method allows to detect sensitively low-concentrations of amide protons in diffuse gliomas.

## Supporting Information

S1 Table The measurements of the MTR_asym_ (3.5 ppm) ΔMTR_asym_ (3.5 ppm) by the two observers. (DOCX)
