# Supplementary material for: Amide Proton Transfer Imaging of Diffuse Gliomas: Effect of Saturation Pulse Length in Parallel Transmission-Based Technique
Source: PLoS One. 2016 May 26;11(5):e0155925. doi: 10.1371/journal.pone.0155925 (PMC4881971; doi:10.1371/journal.pone.0155925)
Supplement: S1 Table — (DOCX) [file pone.0155925.s001.docx]

Observer 1

| LGG | MTR_asym_(3.5ppm)　(%) | | | ΔMTR_asym_(3.5ppm) (%) | | |
| --- | --- | --- | --- | --- | --- | --- |
| Saturation length | 500 ms | 1 s | 2 s | 500 ms | 1 s | 2 s |
| Patient #1 | 1.696 | 1.696 | 1.628 | -0.16 | 0.793 | 1.364 |
| Patient #2 | 2.639 | 2.639 | 1.425 | 0.858 | 0.497 | 1.037 |
| Patient #3 | 2.621 | 2.621 | 1.833 | 0.959 | 1.386 | 1.571 |
| Patient #4 | 1.871 | 1.871 | 2.601 | 0.096 | 1.356 | 2.028 |
| Patient #5 | 2.149 | 2.149 | 2.628 | 0.4 | 1.565 | 2.118 |
| Patient #6 | 0.794 | 0.794 | 2.071 | -0.509 | 0.568 | 2.035 |
| Patient #7 | 1.863 | 1.863 | 2.558 | 1.024 | 2.132 | 2.386 |
| Patient #8 | 1.763 | 1.763 | 1.307 | 1.048 | 1.323 | 1.797 |
| Patient #9 | 1.702 | 1.702 | 2.778 | 0.587 | 1.856 | 2.627 |
| HGG | MTR_asym_(3.5ppm)　(%) | | | ΔMTR_asym_(3.5ppm) (%) | | |
| Saturation length | 500 ms | 1 s | 2 s | 500 ms | 1 s | 2 s |
| Patient #1 | 2.751 | 3.453 | 3.453 | 0.96 | 2.361 | 3.11 |
| Patient #2 | 3.256 | 3.342 | 3.342 | 1.866 | 2.488 | 2.505 |
| Patient #3 | 3.297 | 4.138 | 4.138 | 1.808 | 3.183 | 4.051 |
| Patient #4 | 2.447 | 3.052 | 3.052 | 1.232 | 2.429 | 3.034 |
| Patient #5 | 3.768 | 4.013 | 4.013 | 2.432 | 3.037 | 4.396 |
| Patient #6 | 3.301 | 3.654 | 3.654 | 1.945 | 2.949 | 4.355 |
| Patient #7 | 3.269 | 4.218 | 4.218 | 2.155 | 3.126 | 4.229 |
| Patient #8 | 1.829 | 2.631 | 2.631 | 0.621 | 2.057 | 2.834 |
| Patient #9 | 3.443 | 4.585 | 4.585 | 2.367 | 3.617 | 4.758 |
| Patient#10 | 2.609 | 3.455 | 3.455 | 1.385 | 2.866 | 3.319 |
| Patient#11 | 3.47 | 5.134 | 5.134 | 1.88 | 3.747 | 4.938 |
| Patient#12 | 3.007 | 3.795 | 3.795 | 1.69 | 2.77 | 3.07 |
| Patient#13 | 3.665 | 4.305 | 4.305 | 2.013 | 3.076 | 5.135 |

LGG, low-grade glioma; HGG, high-grade glioma, MTR_asym_ (3.5ppm), magnetization transfer asymmetry at 3.5 ppm.

Observer 2

| LGG | MTR_asym_(3.5ppm)　(%) | | | ΔMTR_asym_(3.5ppm) (%) | | |
| --- | --- | --- | --- | --- | --- | --- |
| Saturation length | 500 ms | 1 s | 2 s | 500 ms | 1 s | 2 s |
| Patient #1 | 3.171 | 2.611 | 2.472 | 1.315 | 1.34 | 2.208 |
| Patient #2 | 2.587 | 2.009 | 1.023 | 0.806 | 0.676 | 0.635 |
| Patient #3 | 2.729 | 2.646 | 1.898 | 1.067 | 1.482 | 1.636 |
| Patient #4 | 1.908 | 2.813 | 2.694 | 0.133 | 1.561 | 2.121 |
| Patient #5 | 2.352 | 2.437 | 2.686 | 0.603 | 1.409 | 2.176 |
| Patient #6 | 0.937 | 1.743 | 2.197 | -0.283 | 0.936 | 2.179 |
| Patient #7 | 1.985 | 2.595 | 2.585 | 1.182 | 2.347 | 2.587 |
| Patient #8 | 1.975 | 1.469 | 1.388 | 1.292 | 1.425 | 1.78 |
| Patient #9 | 1.665 | 2.234 | 3.000 | 0.674 | 1.693 | 3.048 |
| HGG | MTR_asym_(3.5ppm)　(%) | | | ΔMTR_asym_(3.5ppm) (%) | | |
| Saturation length | 500 ms | 1 s | 2 s | 500 ms | 1 s | 2 s |
| Patient #1 | 2.627 | 3.686 | 3.943 | 1.081 | 2.786 | 3.879 |
| Patient #2 | 3.453 | 3.723 | 3.135 | 1.233 | 2.144 | 1.556 |
| Patient #3 | 3.343 | 4.42 | 3.934 | 2.419 | 3.816 | 3.878 |
| Patient #4 | 2.415 | 2.916 | 3.241 | 0.587 | 1.673 | 2.591 |
| Patient #5 | 4.052 | 4.407 | 5.769 | 2.915 | 3.597 | 5.754 |
| Patient #6 | 3.533 | 3.461 | 4.619 | 2.1 | 2.689 | 4.946 |
| Patient #7 | 3.238 | 4.225 | 4.587 | 2.363 | 3.201 | 4.104 |
| Patient #8 | 1.805 | 2.696 | 2.865 | 0.866 | 1.972 | 2.621 |
| Patient #9 | 3.389 | 4.347 | 4.59 | 2.402 | 3.173 | 3.416 |
| Patient#10 | 3.007 | 2.943 | 3.736 | 1.786 | 2.645 | 3.331 |
| Patient#11 | 3.545 | 4.611 | 5.166 | 2.48 | 3.655 | 4.463 |
| Patient#12 | 3.054 | 3.81 | 3.504 | 1.181 | 2.68 | 2.771 |
| Patient#13 | 4.413 | 4.674 | 4.347 | 3.346 | 3.554 | 4.076 |

LGG, low-grade glioma; HGG, high-grade glioma, MTR_asym_ (3.5ppm), magnetization transfer asymmetry at 3.5 ppm.
